# Integrated Assessment of Environment and Health: America’s Children and the Environment

**DOI:** 10.1289/ehp.8321

**Published:** 2005-09-21

**Authors:** Amy D. Kyle, Tracey J. Woodruff, Daniel A. Axelrad

**Affiliations:** 1 School of Public Health, University of California, Berkeley, California, USA; 2 U.S. Environmental Protection Agency, San Francisco, California, USA; 3 U.S. Environmental Protection Agency, Washington, DC, USA

**Keywords:** child, child welfare, children’s environmental health, environmental contaminants, environmental exposure, environmental health, environmental health indicators, environmental health framework, environmental pollutants, integrated assessment

## Abstract

The significance of the environment for health is increasingly being recognized. There is a need for systematic approaches to assessment of environmental factors most relevant to health, health outcomes most influenced by the environment, and the relationships between them, as well as for approaches to representing the results of such assessments in policy deliberations. As a step in the development of such methods, we used findings and data from the environmental protection and public health sectors to develop a set of measures representing topics relevant to children’s environmental health. We used a definition of the environment that emphasized contaminants and a process that involved both analytic and deliberative elements. The steps in this process were to *a*) develop a conceptual framework to depict relationships between environment and health with relevant types of data and information, *b*) select topic areas of significance for children, *c*) identify best available data sources and devise measures, *d*) assess possible surrogate data sources and measures when needed, *e*) design and implement metrics for computation of measures using specified data elements, *f*) select graphical representations of measures, *g*) identify related measures, and *h*) identify data gaps. Representatives of policy and stakeholder audiences participated in this process. The measures are presented in three groups that reflect contaminants in the environment, contaminants in human tissues, and diseases and disorders. The measures present scientifically based representations of data understandable to stakeholders and policy makers that integrate key information from the health and environment sectors in a consistent format.

The significance of environmental factors to the health and well-being of human populations is increasingly apparent [Pew Environmental Health Commission 2000; Rosenstock 2003; World Health Organization (WHO) 1997]. Environmental factors are known or suspected to contribute to important chronic diseases for which incidence has increased, including asthma (Mannino et al. 1998), certain cancers (Ries et al. 1999), and neurodevelopmental outcomes (Blaxill 2004; Landrigan et al. 2002; Mendola et al. 2002; Schettler 2002; Stein et al. 2002).

In the United States, an environmental public health tracking initiative to develop capacity for ongoing assessment of environmental hazards, exposures, and health outcomes is being coordinated by the Centers for Disease Control and Prevention (CDC) (CDC 2003a; Marmagas et al. 2003; McGeehin et al. 2004). This initiative is one example of efforts to better assess, characterize, and address relationships between environmental factors and health and to address the challenges of noninfectious agents and chronic diseases. Initiatives to assess environmental factors that contribute to health status require findings, data, and expertise from both the environmental protection and public health sectors [California Policy Research Center (CPRC) 2004; Institute of Medicine (IOM) 1988]. Integrated assessments use findings and data from different disciplines to generate more informative assessments relevant to public policy problems (Parson 1995). Integrated assessment methods relevant to climate change (Mastrandrea and Schneider 2004; McMichael 1997; Parson et al. 2003; Parson and Fishervanden 1997) and integration of human and ecological risk assessment (Suter 2004) have been developed. Elements of these methods can be applied to environmental health.

To communicate effectively to stakeholders and policy audiences requires development of understandable and interpretable ways to present data. Environmental health indicators are increasingly being used to summarize technical information and characterize key environmental factors, health outcomes, and relationships between them [Briggs et al. 1996; California Department of Health Services (CDHS) 2002; von Schirdning 2002; WHO 2002, 2003]. Such environmental health indicators can be distinguished from indicators that focus primarily on either the environment (U.S. Environmental Protection Agency (EPA) 2003] or on health (Federal Interagency Forum on Child and Family Statistics 2004).

Environmental factors that affect children may differ from those most relevant to adults because children can be both more vulnerable and more highly exposed than adults [National Research Council (NRC) 1993; Tamburlini et al. 2002]. Lifelong consequences of exposures in early life are beginning to be observed (Forrest and Riley 2004; NRC 2004). Efforts to assess children’s environmental health systematically are beginning internationally (Briggs 2003; North American Commission for Environmental Cooperation 2002; Secretariat of the Commission for Environmental Cooperation of North America 2003; United Nations 2002). For example, the WHO in Europe has developed estimates of children’s disease burden from air pollution, water and sanitation, lead, and injury (Valent et al. 2004). Addressing children’s health needs, including those associated with environmental factors, requires targeted approaches to information gathering and assessment (NRC 2004).

In 1999, we began to develop a set of measures relevant to children’s environmental health in the United States. The goals were to *a*) identify environmental contaminants significant for children and diseases or disorders of children likely to be related to environmental contaminants or conditions, *b*) develop quantifiable measures of changes in these contaminants or diseases in the United States for the period 1990 to 2000 using existing data, *c*) assess differences by race/ethnicity and socioeconomic status (SES), *d*) identify areas in need of attention or further research, and *e*) identify data gaps. Initial results were released in 2000 (Woodruff et al. 2000), and an expanded assessment, titled *America’s Children and the Environment: Measures of Contaminants, Body Burdens, and Illnesses*, was released in 2003 (Woodruff et al. 2003). In this article, we report on the framework and methods used to develop this first integrated assessment of environment and health for children in the United States.

## Methods and Approach

**Figure 1:**
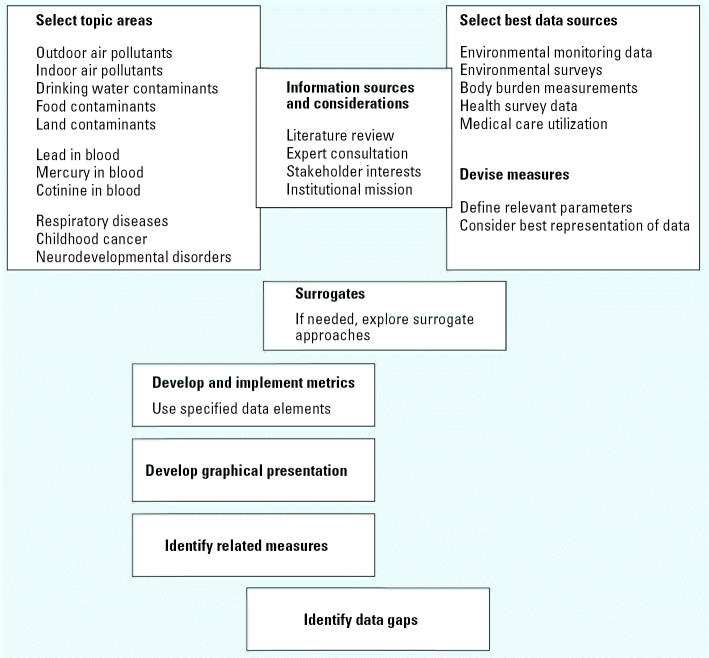
Steps involved in developing the measures of children’s environmental health, after a conceptual model is specified. The first step, development of a conceptual framework, is shown separately in Figure 3.

The steps in the assessment of children’s environmental health, shown in Figure 1, were to develop a framework to represent relationships between environmental factors and health; select topic areas; identify, assess, and select data sources and develop specific measures to represent the data; investigate surrogate measures when data were not available for a measure identified as most directly relevant; specify computational approaches or metrics and data elements to generate the measures and implement them; develop graphical representations of the measures; identify measures that are related; and identify data gaps and future directions for additional research and analysis. Assessment of differences by SES and by race/ethnicity was a critical component, because identifying such differences and looking for their causes is essential to eliminating health disparities.

Our working definition of the “environment” generally encompassed environmental factors or agents subject to management and regulatory attention by the U.S. EPA, the entity that sponsored the project. Use of this working definition represents a step in the development of an approach to assessment of children’s environmental health. It would also be appropriate to use a broader definition of the environment and include elements of the built environment or factors originating in sectors such as education, housing, or transportation.

We convened workshops that included stakeholders and experts in toxicology, epidemiology, children’s health, exposure assessment, and public health surveillance to discuss conceptual approaches, topics to be addressed, data sources, metrics, graphical representations, and data gaps. We consulted with technical and policy experts from key federal agencies. This analytic–deliberative process allowed us to meld the views of technical experts and stakeholders into a consistent approach and to identify the best available data sources and methods to address questions of interest.

### Develop framework to depict the relationship between environment and health.

**Figure 2:**
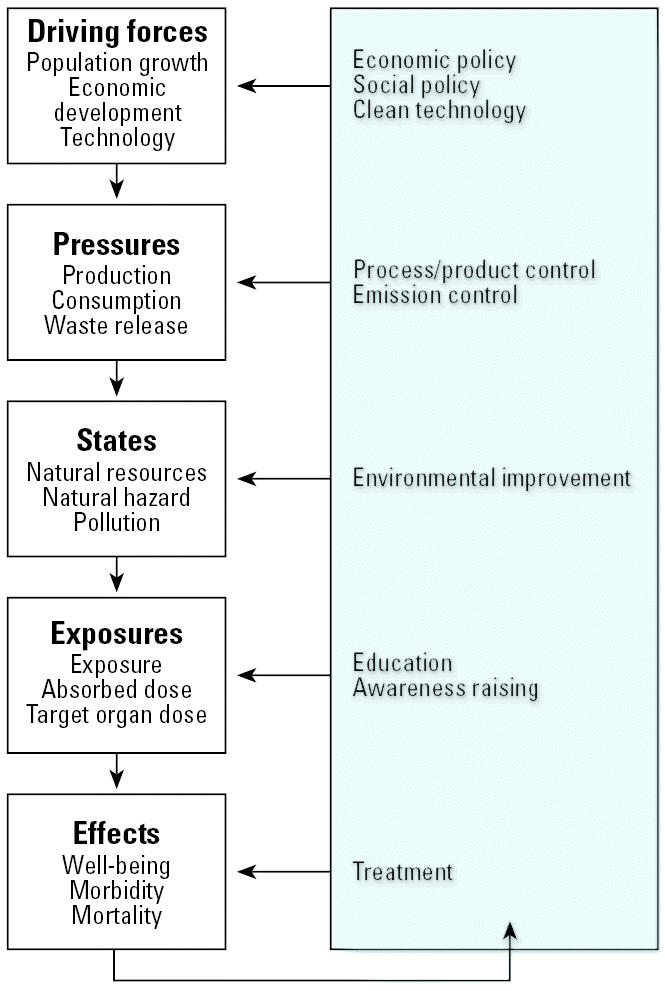
WHO framework for assessment of relationships between environment and health and policy actions or interventions. The DPSEE (driving force, pressure, state, exposure, effect) model is commonly used in international contexts as a framework for developing indicators and assessing relationships between environmental factors and health outcomes. Adapted from a presentation of the model in a recent document from the World Health Organization (Briggs 2003).

We developed a framework to depict relationships between environmental factors and health. We incorporated some elements of a widely used WHO model, which includes: driving forces → pressures → environmental states → exposures → health conditions or effects, shown in Figure 2 (Briggs et al. 1996; Furgal and Gosselin 2002; von Schirdning 2002). Driving forces include major social and economic changes and practices such as urbanization, poverty and inequality, scientific and technical advances, and patterns of production and consumption. Pressures include sources or releases of environmental agents. Environmental states include conditions of environmental media such as lakes or streams.

**Figure 3:**
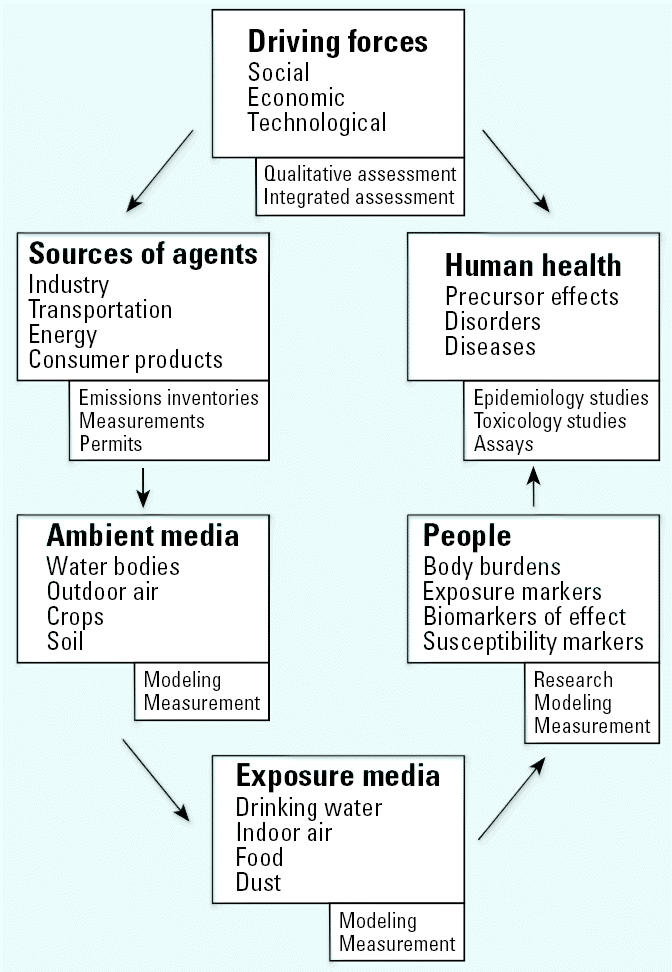
Conceptual framework to represent relationships between environmental factors and health. This framework shows relationships as well as the types of data that can be used to represent the characteristics relevant to each of the major components (shown in the small boxes).

Our framework, shown in Figure 3, includes driving forces; sources of releases of environment agents of concern; concentrations of environmental agents of concern measured or estimated in environmental ambient or exposure media; concentrations of agents of concern in human tissues; and health outcomes (diseases and disorders) in populations. We included driving forces and sources of agents in the framework because control or elimination of sources is the policy strategy that reflects primary prevention. However, we did not develop measures for them because of resource limitations. We do not use the terms “pressures,” “states,” or “responses” because we have found them ambiguous.

Figure 3 shows types of information relevant to each component. Ambient environmental media include outdoor air, water, soil, or agricultural products; exposure media include outdoor air, indoor air, drinking water, food products, and dust. Concentrations in ambient media are often significant determinants of exposure. For example, epidemiologic studies have measured pollutant contaminants in ambient media and quantified relationships to health effects (i.e., relationships between outdoor measurements of fine particulate matter and mortality). In this approach, we consider data about concentrations of environmental agents in exposure media and concentrations of agents of concern in human tissues.

### Identify topic areas to address.

The second step was to identify topic areas of interest. For environmental contaminants, these areas included outdoor air pollutants, indoor air pollutants, drinking water contaminants, contaminants in foods, and contaminants in soil. For contaminants in humans, we included topic areas identified as a concern in the environment and for children for which we could produce a meaningful interpretation of data available from the nationally representative sample developed by CDC (2003b). For diseases and disorders, we included examples important to the health of children for which there was also published research that showed an established or suggested link to one or more environmental contaminants, based on previous analysis, consultation with experts, survey of the scientific literature, and use of standard references and existing reviews (Woodruff et al. 2004). We reviewed emerging research on the links between air pollutants and respiratory outcomes in children and adults, evidence for environmental factors that contribute to cancer in children, and studies that examined links between environmental exposures and neurodevelopmental disorders (Woodruff et al. 2003).

We did not attempt at the outset to identify all topic areas that might be relevant; rather, we endeavored to identify a scope of work that could be accomplished with available resources. We identified agents and outcomes of concern first and then sought data sources for these agents and outcomes to allow for identification of data gaps.

### Assess and select data sources and develop measures.

For each topic area, we concurrently identified and assessed potential data sources and considered relevant ways to represent data. For each candidate data source, we assessed accessibility, validity and reliability, data elements, time period for which data were available, geographic area and resolution, and applicability to children. We sought data sources with sufficient documentation, standard collection procedures, and quality assurance. We consulted key references and knowledgeable parties. When multiple sources were available, we selected the source with the best representation of the United States and best coverage of the study period. For some topic areas, we could not identify usable data sources.

In conjunction with the review of data sources, we developed measures for the topic areas. We reviewed measures included in Healthy People 2010 (U.S. Department of Health and Human Services 2000). In some cases, we concluded that more than one measure was needed. For example, for criteria air pollutants, we included one measure that reflected air quality on a daily basis, which is related to health effects associated with short-term, high concentrations of pollutants. Because chronic exposures to lower concentrations of pollutants are also relevant, we included a measure based on annual concentrations for some pollutants. To reflect the coverage of data sources, we estimated the percent of the population represented.

### Investigate surrogates where data are not available.

If a data source directly representative of a condition of interest was not available, we investigated surrogates that reflected related conditions. For example, we used reported violations of drinking water standards as a surrogate for concentrations of contaminants in drinking water. We assessed data for surrogate measures using the same approach used for other sources.

### Specify computational approach and data elements and implement the measure.

The sixth step was to devise the method to be used to compute or generate the measure, to select the metric, and to identify data elements to be used and their sources. Measures were then computed.

### Design graphical representation of the measure.

Along with the computation of the measure, we selected an approach to present results graphically for each measure. We considered how to show limitations, distributions, and coverage of the data. When possible, presentations showed trends over time and differences by race/ethnicity and SES.

### Identify related measures.

To highlight relationships between contaminants and outcomes, we identified measures that were related. For example, measures that reflect concentrations of mercury in foods would be related to measures that reflect concentrations of mercury in blood of women of childbearing age. Table 1 shows measures that may be viewed as related. Related measures can be considered together to look at patterns with regard to time, geography, race/ethnicity, and SES. This approach can identify additional areas for research, needs for further review or consideration of existing research, or areas in need of policy development or intervention.

### Identify data gaps.

The last step was to describe data gaps. In some cases, we included a narrative description of the topic area as an emerging issue. Other topic areas were identified as data gaps. For even the best data sources, there are usually limitations on coverage or representativeness. We addressed some of these issues in the final step. There are many important topics for children’s environmental health with little or no coverage in the set of measures assembled.

## Results and Discussion

The analysis resulted in the development of measures for environmental contaminants, human body burdens, and diseases and disorders. Table 2 shows the full set of measures and their coverage.

The development of measures raises numerous issues. One issue for environmental contaminant and body burden measures is whether a point of comparison should be used. Measured or estimated values can be compared to regulatory standards, such as ambient air quality standards, or other benchmarks. Such comparisons can be useful because most people understand that concentrations that exceed such standards may be related to potential for disease. However, regulatory standards may result from balancing of health with other factors, such as cost or technologic feasibility of control technologies. Such standards would not represent an appropriate point of comparison from a health perspective. Comparison to a fixed standard can create an impression that there is a “safe” concentration below which exposures would not pose any risk to health. However, for many pollutants, there may be no threshold, as is the case for particulate matter, ozone, and blood concentrations of lead (American Academy of Pediatrics Committee on Environmental Health 2004; Canfield et al. 2003a, 2003b; Lanphear et al. 2000; McMichael et al. 1988; Schwartz 1994).

How to reflect the distribution of the data is important as well. For example, for blood lead concentrations, the median or average value gives an idea of the typical child’s exposure, but will not convey the potential magnitude of risk that could be experienced by children with concentrations at the higher end, such as the 95th percentile. It is useful to report both central and high-end estimates and to characterize groups likely to be affected by the higher exposures. This approach may be important for identifying health disparities or differences in exposures.

The analysis identified numerous data gaps. For criteria air pollutants, a significant gap is the geographic extent of the monitoring network. Even when monitors are assigned by county, many counties have no data. This data gap might be rectified best by additional modeling. For hazardous air pollutants, the assessment was based on model predictions of ambient concentrations of a certain number of hazardous air pollutants. There are two structural limitations for this data source. One is that the modeling is done only every 3 years, and the results are presented several years after the year to which they apply. The second is that the approach includes only a relatively small number of pollutants.

For indoor air pollutants, data do not exist on any large scale. Different approaches to assessing indoor air pollutants and indoor environments as a whole are needed. We believe that surrogate measures will be necessary for indoor pollutants.

For drinking water contaminants, the national data reporting system has the significant limitation that violations, not measured concentrations, are reported. The latter would be more informative, but such data are available only at the state level. There are also significant limitations on monitoring and reporting.

For food and land contaminants, the data available are very limited. Surrogates were needed in both categories. Substantial additional assessment would be needed to characterize these areas fully.

For body burdens, the data available for most contaminants come from the recent monitoring programs developed by the CDC. Because this initiative is relatively new, the data are limited to only a few years.

For diseases, surveys such as the National Health Interview Survey provides a good picture of the population as a whole, but it does not allow for breakout by geographic area or state. The information cannot be put on a common scale with other environmental data or information. For some important health outcomes, such as birth defects, there is no national data source that can be used. Data for neurodevelopmental effects are also very limited.

What to include in an assessment is an important consideration. The working definition of “the environment” used for these measures corresponded closely to the mandates of the U.S. EPA. It included environmental agents that can contaminate environmental media resulting in exposure. Such agents fall under regulatory mandates of the U.S. EPA. However, many other factors can be viewed as falling under the rubric of the environment. It may be more difficult to identify data sources if a more expansive definition of environmental factors is used in future work. Even with this relatively narrow scope, there are significant limits to our understanding of the links between environmental factors and health outcomes. In conducting an assessment that is geared to reporting progress and identifying areas in need of attention, it is important to consider probable contributors to disease and diseases that are likely caused at least partly by environmental factors, even when these relationships have not been fully established.

It is helpful to look at available information in two ways. It is beneficial to look at toxicology and other experimental results, to see what can be learned about possible relationships of environmental factors to health outcomes or related biologic effects. Such literature will be available for compounds that have not been included in epidemiologic studies, including agents for which widespread human exposure has not yet occurred or has not yet been measured. Conversely, it is useful to consider results of epidemiologic studies that identify environmental factors that contribute to disease, recognizing that such studies can be conducted only after significant human exposure has occurred.

Defining the type of data appropriate to assess components of a conceptual framework is an important step. The commonly used terms “hazard” and “exposure” represent general concepts rather than particular approaches to measurement. “Hazard” has been used to refer to several different types of data, including those that reflect production, uses, releases, concentrations in environmental media, and concentrations in exposure media of chemicals. All of these types of data can be important, but they also provide different types of information that can be explicated more carefully. Types of “hazard” metrics need to be defined better, and distinctions must be clarified.

Using measures that address different parts of the framework can be informative. Ideally, increasing trends in concentration of environmental contaminants or body burdens would lead to further investigation and policy action aimed at reversing the trend. Monitoring trends in illnesses that are both known and suspected of being associated with environmental factors is important, given the limitations of scientific knowledge of relationships between environmental factors and diseases. Increasing trends in illnesses also are worthy of attention and action to identify and address possible causal factors.

Work that focuses on children’s environmental health has led to the development of the Multiple Exposure–Multiple Effects (MEME) model (Briggs 2003), which emphasizes the multiple relationships between environmental factors and health outcomes. A single environmental agent or factor may contribute to multiple health outcomes, and a single outcome may be affected by multiple environmental factors. How to address the genuine complexity posed by these “many-to-many” relationships remains an important question. There are different ways in which linkages between environment and health can be conceptualized and implemented. Because of the multiple relationships between many environmental factors and health outcomes, it would be enormously complex to model all relationships or to represent the results of such a model. However, it is possible to synthesize and present available data in ways that identify environmental factors relevant to health and diseases or disorders with possible or likely environmental causes and to show likely relationships in ways that are cognizant of the “many-to-many” nature of these relationships.

For future work, it is important to consider what determinants of exposure can be systematically tracked on a large scale. Exposure of individuals cannot be easily monitored or tracked on a large scale partly because individuals’ actions mediate it. Determinants can be further understood through use of models that integrate environmental determinants of exposure with behavioral determinants of exposure, to provide useful data for understanding the relationship between environment and health.

Further development of a concept of determinants of population exposure is needed, along with research to better identify these determinants. Much of the assessment work conducted in environmental health relates to estimation of exposure and consequent doses of environmental contaminants for individuals, as well as research on the relationships between such exposures or doses and adverse health outcomes. Such work establishes understanding of the relationships between environmental factors and health. However, the primary goal is not to establish such relationships. Rather, it is to identify and track the element that contribute to exposure and to adverse health outcomes on a broad scale in ways that are informative to stakeholders and policy communities. The purpose is to identify needs for specific actions to improve health. In this context, it is the determinants of exposure that are, in most cases, going to be amenable to measurement or estimation on a broad scale and also to intervention. Further, analysis of such determinants is critical to better linkage between assessment and intervention.

Because the purposes of tracking or integrated assessment are to improve public health and reduce environmental factors that contribute to disease, consideration of the needs of stakeholders and policy makers who are in a position to take the necessary actions is a key priority from the outset. This work represents a beginning to develop such methods, but more needs to be done.

It would also be relevant to consider administrative or policy actions that contribute to the various environmental conditions portrayed. So, for example, permit requirements for power plants have a bearing on emissions of several key air pollutants. Such “administrative” measures could be developed to address these concerns, and this process would more directly link results to policy change or evaluation.

An integrated assessment can provide a framework to portray diverse data sources to reflect key elements that affect environmental health status. It may rely on data generated for a variety of purposes and adapted to forms that can reflect the purposes of the assessment. Additional challenges include further development of data sources and measures to address some of the key data gaps; to strengthen the measures for driving forces, sources, and other sectors; to explore the implications of the MEME models; to elucidate better the relationship between links in the chain from environment to health; and to identify policy approaches that could reduce the determinants of ill health and promote determinants of good health.

## Figures and Tables

**Table 1:** Measures that may be viewed as related.

Environmental contaminants	Body burdens	Diseases or disorders
Outdoor air pollutants: criteria pollutants		Respiratory illnesses
Outdoor air pollutants: lead Special features: lead in schools Drinking water: lead violations	Blood lead concentrations	Neurodevelopmental disorders
Indoor air pollutants: smoking in homes	Cotinine (marker of tobacco smoke exposure) in blood	Respiratory illnesses
Pesticides detected in foods	Pesticide use in schools	
Warnings of methylmercury in fish	Mercury in blood of pregnant women	Neurodevelopmental disorders

**Table 2:** Measures in *America’s Children and the Environment* for environmental contaminants, body burdens, and diseases.

Topic area	Description of measure	Time period	Coverage	Geographic resolution	Notes
Measures for environmental contaminants					
Common air pollutants	E1: Percentage of children living in counties in which air quality standards were exceeded	1990–2000	Varies by pollutant^a^	County	Includes ozone, PM_10_, SO_2_, NO_2_, and lead (where data are available)
Common air pollutants	E2: Percentage of children’s days with good, moderate, or unhealthy air quality	1990–2000	Varies by pollutant^a^	County	Includes ozone, PM_10_, SO_2_, NO_2_, and CO (where data are available)
Common air pollutants	E3a: Long-term trends in annual average concentration of common pollutants	1990–2000	Varies by pollutant^a^	County	Includes three common air pollutants with long-term standards: PM_10_, SO_2_, NO_2_
Common air pollutants	E3b: Number of children living in counties with high annual concentrations of PM_10_	1990–2000	About 70% of children	County	
Hazardous air pollutants	E4: Percentage of children living in counties where hazardous air pollutant concentrations exceeded benchmarks	1996	Continental USA	County	1 year only; based on estimates for 33 pollutants
Environmental tobacco smoke	E5: Percentage of homes with children < 7 years of age where someone smokes regularly	1994–1999	U.S. population	National	Based on representative sample of U.S. population. Surrogate for concentrations
Drinking water contaminants	E6: Percentage of children living in areas served by public water systems that exceeded a drinking water standard or violated treatment requirements	1993–1999	About 85% of population	County	Data on violations are incomplete Measure is a surrogate for concentrations of contaminants
Drinking water contaminants monitoring and reporting	E7: Percentage of children living in areas with major violations of drinking water monitoring and reporting requirements	1993–1999	About 85% of population	County	Shows children living in areas without reported data
Food contaminants pesticide use	E8: Percentage of fruits, vegetables, and grains with detectable residues of organophosphate pesticides	1994–2001	From distribution centers in 10 states representing 50% of population	National	Surrogate for dietary pesticide exposure to organophosphate pesticides
Land contaminants hazardous waste sites	E10: Percentage of children residing within 1 mile of a Superfund site	1990–2000	All Superfund sites	Site specific locations	Does not reflect sites not included on National Priority List. Surrogate for exposure
Measures for body burdens					
Lead in blood	B1: Concentration of lead in blood of children ≤ 5 years of age	1976–2000	U.S. population	National	Based on representative sample of U.S. population
Lead in blood	B2: Median concentrations of lead in blood of children 1–5 years of age, by race/ethnicity and family income	1999–2000	U.S. population	National	Based on representative sample of U.S. population
Lead in blood	B3: Distribution of concentrations of lead in blood of children 1–5 years of age	1999–2000	U.S. population	National	Based on representative sample of U.S. population
Mercury in blood	B4: Distribution of concentrations of mercury in blood of women of child-bearing age	1999–2000	U.S. population	National	Based on representative sample of U.S. population
Cotinine in blood	B5: Concentrations of cotinine in blood of children	1988–2000	U.S. population	National	Based on representative sample of U.S. population
Measures for childhood diseases and disorders					
Respiratory disease	D1: Percentage of children with asthma	1980–2001	U.S. population	National	Based on representative sample of U.S. population
Respiratory disease	D2: Percentage of children having an asthma attack in the previous 12 months, by race/ethnicity and family income	1997–2000	U.S. population	National	Based on representative sample of U.S. population
Respiratory disease	D3: Children’s emergency room visits for asthma and other respiratory causes	1992–1999	U.S. population	National	Based on representative sample of U.S. population
Respiratory disease	D4: Children’s hospital admissions for asthma and other respiratory causes	1980–1999	U.S. population	National	Based on representative sample of U.S. population
Cancer	D5: Cancer incidence and mortality for children < 20 years of age	1975–1998	U.S. population	National	Based on representative sample of U.S. population
Cancer	D6: Cancer incidence for children < 20 years of age by type	1974–1998	U.S. population	National	Based on representative sample of U.S. population
Neurodevelopmental disorders	D7: Children reported to have mental retardation, by race/ethnicity and family income	1997–2000	U.S. population	National	Based on representative sample of U.S. population

Designations E, B, and D, are from the original report (Woodruff et al. 2003).

a Ozone, about 80% of children; PM_10_ (particulate matter < 10 μm in aerodynamic diameter), about 70% of children; sulfur dioxide (SO_2_), about 50% of children; CO, about 45% of children; nitrogen dioxide (NO_2_), about 50% of children; lead, about 40% of children.
